# Association of Structural Forms of 17q21.31 with the Risk of Progressive Supranuclear Palsy and *MAPT* Sub‐haplotypes

**DOI:** 10.1002/alz.086923

**Published:** 2025-01-03

**Authors:** Hui Wang, Timothy S Chang, Beth A. Dombroski, Po‐Liang Cheng, Ya‐Qin Si, Albert A Tucci, Li‐San Wang, Jung‐Ying Tzeng, Daniel H. Geschwind, Gerald D. Schellenberg, Wan‐Ping Lee

**Affiliations:** ^1^ Department of Pathology and Laboratory Medicine, Perelman School of Medicine, University of Pennsylvania, Philadelphia, PA USA; ^2^ Penn Neurodegeneration Genomics Center, Perelman School of Medicine, University of Pennsylvania, Philadelphia, PA USA; ^3^ Movement Disorders Programs, Department of Neurology, David Geffen School of Medicine, University of California, Los Angeles, Los Angeles, CA USA; ^4^ Bioinformatics Research Center, North Carolina State University, Raleigh, NC USA; ^5^ Penn Neurodegeneration Genomics Center, University of Pennsylvania Perelman School of Medicine, Philadelphia, PA USA; ^6^ Department of Pathology and Laboratory Medicine, University of Pennsylvania Perelman School of Medicine, Philadelphia, PA USA; ^7^ David Geffen School of Medicine, University of California, Los Angeles, Los Angeles, CA USA

## Abstract

**Background:**

The H1/H2 haplotype on 17q21.31 represent the foremost genetic factor contributing to the risk of progressive supranuclear palsy (PSP). Various structural forms of 17q21.31 characterized by the number of copies of α, β, and γ duplications have been identified. However, the specific effect of each structural form on the risk of PSP has never been evaluated in a large cohort study.

**Method:**

Whole genome sequencing data of 1,684 (1,386 autopsy confirmed) individuals with PSP and 2,392 control subjects were obtained from the Alzheimer’s Disease Sequencing Project (ADSP) Umbrella NG00067.v7. Copy numbers of α, β and γ were called by CNVpyter (Version 1.3.1). Expectation‐maximization algorithm was used to infer the sturctural forms of 17q21.31 for each individual. Then, a case‐control study was conducted to investigate the association of copy numbers of α, β, and γ duplications and structural forms of 17q21.31 with the risk of PSP.

**Result:**

The copy number of γ was independently associated with the increased risk of PSP. Each additional duplication of γ was associated with 1.10 (95% CI, 1.04‐1.17; *P* = 0.0018) fold of increased risk of PSP when conditioning H1 and H2. For H1 haplotype, addition γ duplications displayed a higher odds ratio for PSP: the odds ratio increases from 1.21 (95%CI 1.10‐1.33, *P* = 5.47 × 10^‐5^) for H1β1γ1 to 1.29 (95%CI 1.16‐1.43, *P* = 1.35 × 10^‐6^) for H1β1γ2, 1.45 (95%CI 1.27‐1.65, *P* = 3.94 × 10^‐8^) for H1β1γ3, and 1.57 (95%CI 1.10‐2.26, *P* = 1.35 × 10^‐2^) for H1β1γ4. Moreover, H1β1γ3 is in linkage disequilibrium with H1c (R^2^ = 0.31), a widely recognized *MAPT* sub‐haplotype associated with increased risk of PSP. The proportion of *MAPT* sub‐haplotypes associated with increased risk of PSP (i.e., H1c, H1d, H1g, H1o, and H1h) increase from 34% in H1β1γ1 to 77% in H1β1γ4.

**Conclusion:**

This study identified that the copy number of γ was associated with the risk of PSP. H1 haplotypes with more γ duplication showed a higher odds ratio and were associated with *MAPT* sub‐haplotypes with increased risk of PSP. These findings expand our understanding of how the complex structure of H1/H2 affect the risk of PSP.

